# The contribution of the gut-liver axis to the immune signaling pathway of NAFLD

**DOI:** 10.3389/fimmu.2022.968799

**Published:** 2022-08-31

**Authors:** Jiayi Liu, Anding Wu, Jingjing Cai, Zhi-Gang She, Hongliang Li

**Affiliations:** ^1^ Department of Cardiology, Renmin Hospital of Wuhan University, Wuhan, China; ^2^ Institute of Model Animal of Wuhan University, Wuhan, China; ^3^ Department of general surgery, Huanggang Central Hospital, Huanggang, China; ^4^ Huanggang Institute of Translation Medicine, Huanggang, China; ^5^ Department of Cardiology, The Third Xiangya Hospital, Central South University, Changsha, China; ^6^ School of Basic Medical Sciences, Wuhan University, Wuhan, China; ^7^ Medical Science Research Center, Zhongnan Hospital of Wuhan University, Wuhan, China

**Keywords:** NAFLD (nonalcoholic fatty liver disease), intestinal flora, gut-liver axis, Inflammation, lipid metabolism, insulin resistance

## Abstract

Nonalcoholic fatty liver disease (NAFLD) is the liver manifestation of metabolic syndrome and is the most common chronic liver disease in the world. The pathogenesis of NAFLD has not been fully clarified; it involves metabolic disturbances, inflammation, oxidative stress, and various forms of cell death. The “intestinal-liver axis” theory, developed in recent years, holds that there is a certain relationship between liver disease and the intestinal tract, and changes in intestinal flora are closely involved in the development of NAFLD. Many studies have found that the intestinal flora regulates the pathogenesis of NAFLD by affecting energy metabolism, inducing endotoxemia, producing endogenous ethanol, and regulating bile acid and choline metabolism. In this review, we highlighted the updated discoveries in intestinal flora dysregulation and their link to the pathogenesis mechanism of NAFLD and summarized potential treatments of NAFLD related to the gut microbiome.

## Introduction

The gut-liver axis is the bidirectional relationship between intestinal microorganisms and the liver, which is affected by diet, heredity, the environment, and other factors (1). The intestine and liver originate from the foregut at the embryonic stage. Venous blood carries nutrients absorbed from food, factors from intestinal microbiota, and immunoreactive products into liver tissue through the portal vein. At the same time, bile acids (BAs) synthesized by hepatocytes combine with glycine or taurine to form bile salts in the liver, which are then stored in the gallbladder and eventually enter the small intestine (2). More than 70% of the blood in the liver comes from the intestinal tract and enters the liver through the portal vein.

**Figure 1:**
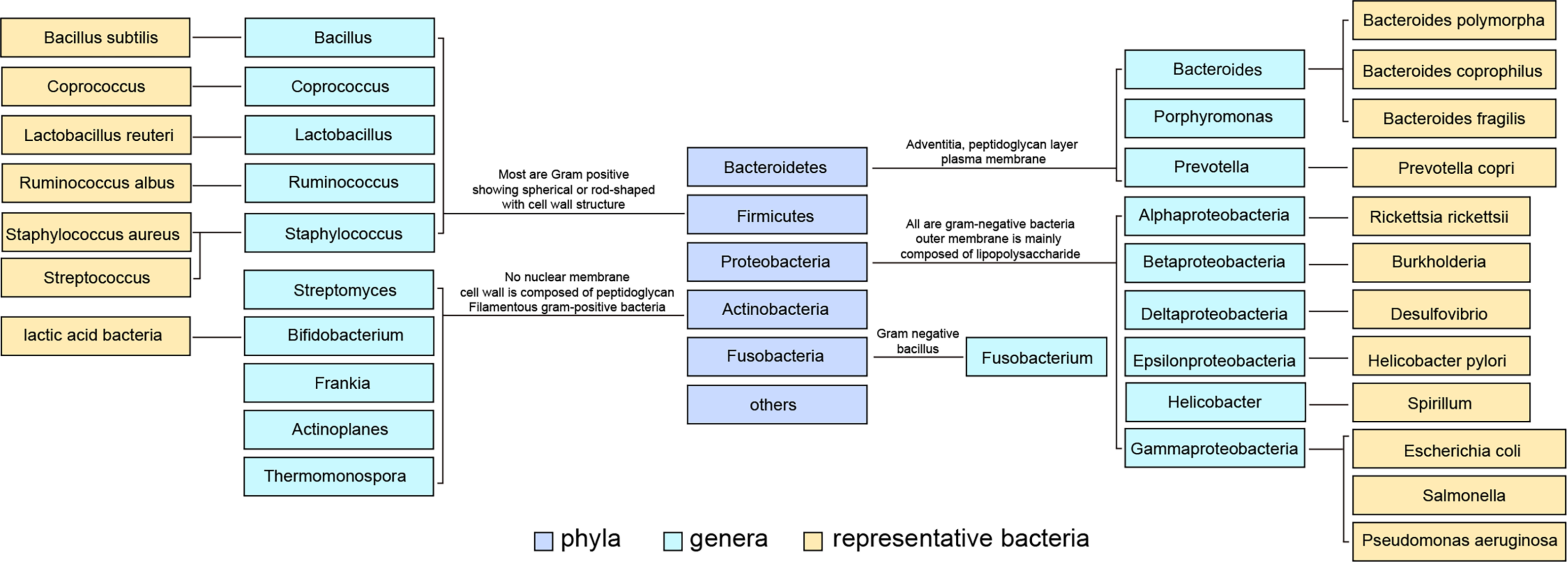
Classification of common intestinal flora and some representative flora. Intestinal bacteria are divided into phyla phylum, class, order, family, genus and species according to their grades. The content of Firmicutes in normal intestinal flora was the highest, and Bacteroidetes ranked second.

The intestinal tract contains a large number of bacteria, which help the human body absorb energy and nutrients. Some toxins and flora products absorbed through the intestinal tract depend on the liver’s metabolism. Among billions of microorganisms in the intestinal flora, there are more than 100 species of bacteria. The contents of *Firmicutes* and *Bacteroidetes* in normal intestinal flora are the highest, accounting for 90% of the total number of bacteria (3) (**Figure 1**). The intestinal mucosal and vascular barrier is the functional and anatomical structure that allows nutrients to access the circulation and reach the liver without dispersing microbes and toxins from the gut. Bacterial outgrowth and composition changes or damage to the intestinal barrier increase microbial exposure and the proinflammatory environment of the liver (4, 5).

The imbalance in intestinal flora usually has the following two characteristics : (1) a decrease or complete loss of some symbiotic flora that leads to a decrease in flora diversity, which is related to many immune responses and metabolic disorders (6); and (2) the overgrowth of pathogenic bacteria. In healthy intestinal ecosystems, the proportion of pathogenic bacteria in intestinal flora is relatively low. However, in many diseases, the growth of pathogenic bacteria exceeds that of other bacteria. For example, the abundance of *Escherichia coli* (a subclass of *Proteobacteria*) increases in many immune inflammatory and metabolic diseases, including NAFLD (7, 8). The proliferation of *Amoeba* is generally considered a potential diagnostic marker of flora imbalance and disease (9).

NAFLD is the most common chronic liver disease in the world and includes a series of liver lesions, from simple steatosis to nonalcoholic steatohepatitis (NASH), cirrhosis, and hepatocellular carcinoma (10). The pathological mechanism of NAFLD is primarily linked to obesity, insulin resistance, and lipid dysregulation. NAFLD is closely raleted to metabolic syndrome and the pathogenesis of which is studied mainly based on metabonomics (11, 12). In recent years, there is increasing evidence showing that the NAFLD related to the imbalance in intestinal flora (13). The latest study, recently published in science translational medicine, provides predictions of long-term NAFLD development based on clinical indicators of NAFLD patients, intestinal flora macrogenomics and metabonomics data (14). In addition, there is also other evidence which indicated that intestinal microbiota affects NAFLD by regulating metabonomics. For instance, the results of clinical metabonomics show that the imbalance of intestinal flora is related to the imbalance of amino acid metabolism in the pathogenesis of NAFLD (15). Amino acid therapy could effectively regulate intestinal microflora and fatty acid oxidation in mice and improve NASH (16).

Although a number of clinical and animal experiments have observed that intestinal flora imbalance is involved in the pathogenesis of NAFLD (17). However, It is still not clear whether the imbalance in intestinal flora is the direct cause of NAFLD or just reflects some disease-related changes in the host immune and metabolic system. In this review, we highlighted the updated discoveries in intestinal flora dysregulation and their link to the pathogenesis mechanism of NAFLD and summarized potential treatments of NAFLD related to the gut microbiome.

## Study on the intestinal microflora in patients with NAFLD/NASH

**Figure 2:**
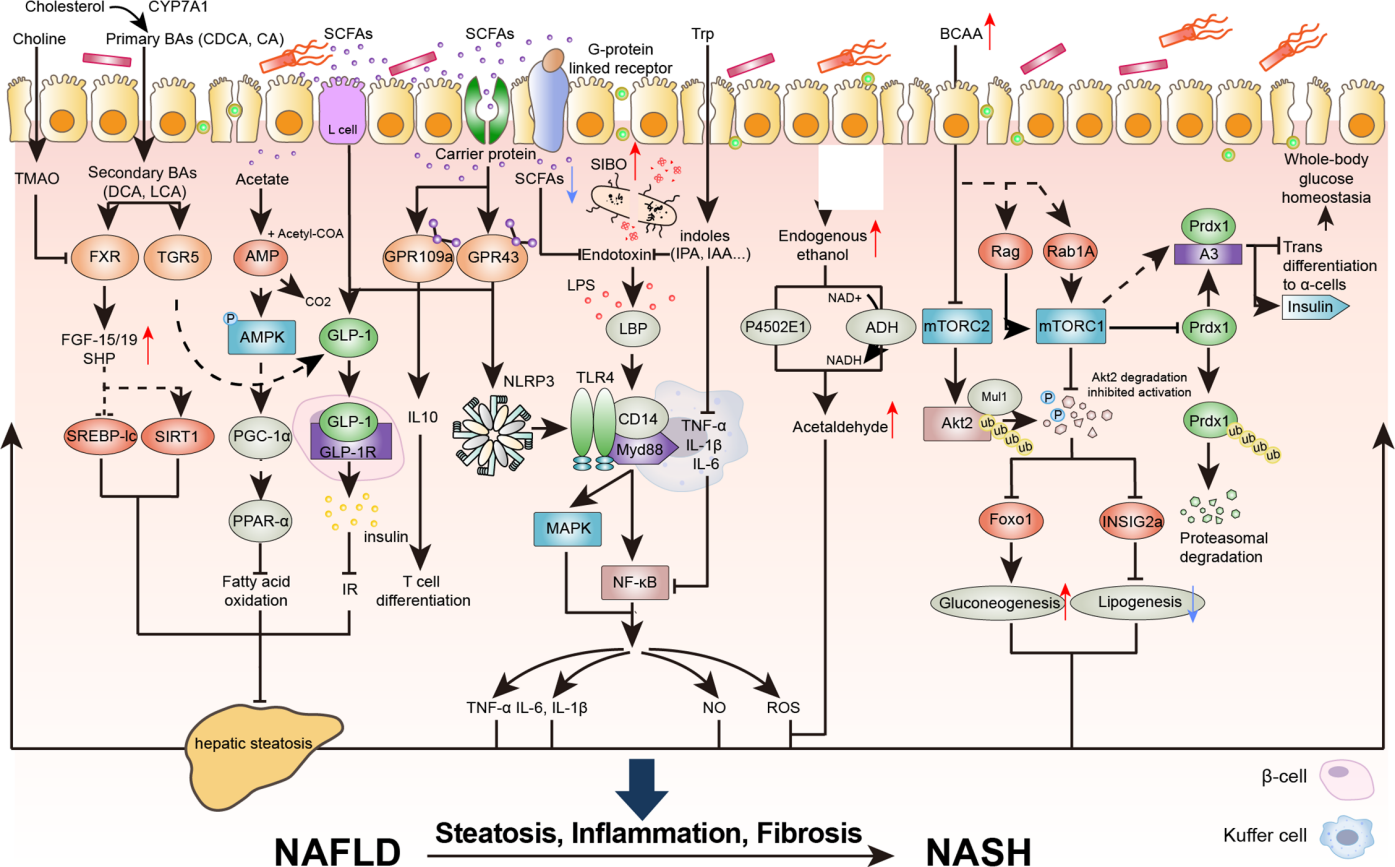
The destruction of the intestinal epithelial barrier caused by intestinal flora imbalance is an important condition for the development of NAFLD and nonalcoholic steatohepatitis (NASH). Intestinal inflammation and the production of metabolic toxins cause intestinal barrier dysfunction, exposing the liver to flora metabolites and promoting the development of NAFLD. BAs, bile acids; CDCA, chenodeoxycholic acid; CA, cholic acid; DCA, deoxycholic acid; LCA, lithocholic acid; TMAO, trimethylamine oxide; TGR5, Takeda G protein-coupled receptor 5; FGF, fibroblast growth factor; SHP, small heterodimer partner; SREBP-lc, sterol regulatory element-binding protein-lc; SIRT1, sirtuin 1; PGC-1a, proliferator activated receptor g coactivator 1 a; PPARa, peroxisome proliferator activated receptor alpha; GLP, glucagon like peptide; GPR, G protein-coupled receptor; TNF, tumor necrosis factor; IL-6, interleukin; NLRP3, NOD-like receptor protein 3; LPS, lipopolysaccharide; LBP, lipopolysaccharidebinding protein; SIBO, Small intestinal bacterial overgrowth; IPA, Indole-3-propionic acid; IAA, 3-Indoleacetic acid; Myd88, myeloid differentiation factor 88; MAPK, mitogen-activated protein kinase; NF-kB, nuclear factor kappa-B; NO, nitric oxide; ROS, reactive oxygen species; Trp, tryptophan; NAD, nicotinamide adenine dinucleotide; BCAA, branched-chain amino acid; mTORC, mammalian target of rapamycin complex; Akt, protein kinase B; INSIG2a, insulin induced gene 2a; Foxo1, forkhead box O1; Mul1, mitochondrial E3 ubiquitin protein ligase 1; RAB1A, member ras oncogene family; Prdx1, peroxiredoxin 1; Ub, ubiquitin; p-, Phosphorylation.

The results of high-throughput sequencing of clinical samples showed that the abundances of *Escherichia coli, Dysgonomonas*, and *Bilophila* increased in patients with NAFLD. These conditional intestinal pathogens promote the production of endotoxin and endogenous ethanol, which increases systemic inflammatory grade and insulin resistance. Moreover, the abundance of beneficial bacteria, such as *Alistipes, Bifidobacterium*, and *Akkermansia muciniphila* decreased, which impaired the production of short-chain fatty acids (SCFAs) to maintain the integrity of the intestinal mucosal barrier and facilitated the proliferation of harmful bacteria and the inflammatory response to promote NAFLD (7, 18–20). Studies have shown that intestinal flora imbalance often exists in patients with chronic liver disease and that the degree of imbalance is positively associated with the severity of liver disease (21, 22). In the following section, we discuss the role of the intestinal flora in the regulation of the immune signaling pathways in the progression of NAFLD (**Figure 2**).

## Key mechanisms involved in the regulation of intestinal microbiota during NAFLD progression

### Intestinal endotoxin

#### Proinflammatory effect

Endotoxin is a complex of lipopolysaccharide (LPS) and trace proteins on the outer membrane of *Helicobacter genus* and Gram-negative bacteria (23). LPS is an active component of endotoxin that migrates into intestinal capillaries through Toll-like receptor (TLR)-dependent channels. Compared with patients with abnormal metabolism without NAFLD, the serum level of LPS was increased in patients with NAFLD (24). A large number of studies have shown that intestinal endotoxin plays an important role in the occurrence and development of NAFLD and that the level is correlated with the severity of NAFLD (24). LPS accelerates systemic and local inflammatory responses to promote NAFLD progression into NASH (8, 25, 26). Endotoxin binds to lipopolysaccharide-binding protein (LBP) in blood, which transfers LPS to Kupffer cells and binds to the cell surface TLR4-CD14 complex to activate downstream mitogen-activated protein kinase (MAPK) and nuclear factor kappa-B (NF-κB) inflammatory signaling pathways (25, 27). The activation of these pathways leads to the activation of proinflammatory factors such as tumor necrosis factor (TNF), (interleukin) IL-6, and IL-1β, as well as bioactive substances such as nitric oxide (NO) and oxygen free radicals, forming a network of inflammatory mediators (28).

Among all LPS ligands, LPS-TLR4 is the primary interactive pair in the progression of NAFLD. In addition, studies in animal models have shown that TLR 2, 5, and 9 are also involved in the development of NAFLD (29). When TLR ligands are stimulated, host cells produce various responses, mainly through four kinds of effector molecules, including myeloid differentiation factor (Myd) 88, Toll-Interleukin receptor domain-containing (TIRA), TIR-domain-containing adapter-inducing interferon (TRIF), and Trif-related adaptor molecule (TRAM), which lead to the activation of NF-κB, interferon (IFN) regulator 3 (1RF-3) and activator protein (AP)-1 (30, 31). The liver is an immune target organ; under normal circumstances, inflammation is not triggered. When excessive intestinal flora and toxins reach the liver and exceed the clearance capacity of the liver, they stimulate inflammatory reactions and aggravate liver injury and even liver fibrosis.

#### Increased intestinal mucosal permeability

In general, endotoxin is considered a useful bacterial biomarker for increased intestinal permeability because it can be transferred from the intestine to the systemic circulation through an incomplete intestinal mucosal barrier (32). It also activates the complement and coagulation systems and promotes macrophage infiltration to further damage intestinal mucosal barrier function directly or indirectly (33). Some studies have suggested that endotoxin damages the local intestinal mucosa and triggers an inflammatory cascade by inhibiting the migration of new intestinal epithelial cells and weakening the repair effect of cell repair factors, eventually resulting in local intestinal mucosal ischemic necrosis and intestinal barrier damage (34).

#### Small intestinal bacterial overgrowth

Small intestinal bacterial overgrowth (SIBO) has been shown to be related to the pathogenesis of NAFLD (35). SIBO mainly refers to the increase in the number of Gram-negative bacteria, the destruction of the tight junction between intestinal epithelial cells, the increase in intestinal mucosal permeability, low-grade endotoxemia and the production of cytokines in Kupffer cells (36, 37).

Studies have found that intestinal endotoxemia plays a particularly prominent role in the environmental factors affecting the occurrence of NAFLD in mice, and the overgrowth of intestinal bacteria aggravates the production of endotoxin (38, 39). In addition, a meta-analysis comprising 10 studies showed that SIBO was significantly correlated with NAFLD, with a combined odds ratio of 3.82 (95% confidence interval, 1.93-7.59%) (40). The rate of SIBO was found to be 37.5% in patients with NAFLD. Compared with the levels in patients without SIBO, the level of endotoxin and the expression of hepatic TLR4 signaling genes were significantly increased in SIBO patients (27).

#### Endogenous ethanol

Compared to simple obesity, there is an increased abundance of alcohol-producing bacteria in NASH microbiomes, including *Proteobacteria, Enterobacteriaceae*, and *Escherichia*, resulting in elevated blood-ethanol concentrations in NASH patients (8) (see **Table 1**). Alcohol-producing bacteria produce endogenous alcohol by fermentation in the intestine, which is absorbed into the liver through the gastrointestinal tract and oxidized to acetaldehyde under the action of alcohol dehydrogenase (ADH; 80%) and cytochrome P4502E1 (20%) in the liver (50). It is then oxidized to acetic acid by aldehyde dehydrogenase (ALDH) and finally enters the tricarboxylic acid cycle to produce carbon dioxide and water. Endogenous alcohol not only directly damages the liver but also damages the liver through its oxidation product acetaldehyde by increasing the production of peroxide and oxidative stress to induce and aggravate the occurrence and development of NASH.

**Table 1:** Metabolite and related flora.

Metabolite	Class	Related flora (producing flora or acting flora)
Intestinal endotoxemia	Endotoxin	*Helicobacter genus, Gram-negative bacterium (* 23)
	endogenous alcohol	*Proteobacteria, Enterobacteriaceae, and Escherichia (* 8)
Amino acid	BCAA	*Rruminococcus*, decrease of *Coprococcus (* 41)
	indole and derivatives (IPA, IAA)	*Bacteroides polymorpha, Clostridium, Enterococcus faecalis* and *Escherichia coli* (42)
SCFAs	acetic acid	Anaerobes (including *Bacteroides, Lactobacillus, Streptococcus* and *Bifidobacterium*) (43)
	propionic acid	*Bacteroidetes* (44)
	butyric acid	*Clostridium, Spirillum, Bacillus and Ruminococcus (* 45, 46)
BAs	/	*Lactobacillus, Bifidobacterium, Clostridium* and *Bacteroides (* 8, 47)
Trimetlylamine oxide	TMA	obligately anaerobic Clostridia (*phylum Firmicutes*) and facultatively anaerobic Enterobacteriaceae (*phylum Proteobacteria*) (48, 49)

Animal experiments have also confirmed that intestinal flora fermenting ethanol from sugars rather than simple fatty liver leads to liver damage and NASH (51). Acetaldehyde, an intermediate metabolite of ethanol, causes direct oxidative damage to the tissue and liver. Acetaldehyde increases the production of oxygen free radicals and lipid peroxidation and causes hepatocyte injury by activating the activities of related enzymes in the body (52). Dunagan M’s study found that acetaldehyde destroys tight junctions between intestinal epithelial cells and increases the permeability of monolayer colon adenocarcinoma cells (Caco-2 cell monolayer) to endotoxin (53). In addition, acetaldehyde induces mitochondrial dysfunction and makes hepatocytes more vulnerable to oxidative damage (54).

### Amino acids

#### Branched-chain amino acids

The intestinal flora affects the host metabolic phenotype through a variety of mechanisms, including fermentation to produce high-energy substrates, especially branched-chain amino acids (BCAAs). When BCAAs were added to the diet, the level of acetic acid-producing *Ruminococcus* increased, and the level of acetic acid in the portal vein increased, thus reducing fat accumulation in the liver (41). The possible mechanism is that the BCAA-mammalian target of rapamycin complex (mTORC) 2 depends on the mitochondrial E3 ubiquitin protein ligase 1 (Mul1) to induce ubiquitination and degradation of protein kinase B 2 (Akt2), which inhibits liver adipogenesis by interrupting Akt2- insulin induced gene 2 a (INSIG2a) signal transduction. In addition, BCAAs regulate Akt2/forkhead box O1 (Foxo1) signal transduction and increase liver glucose production (55). Moreover, BCAAs are also associated with decreased levels of *Coprococcus*, which are closely related to inflammation and might be beneficial to NAFLD (41).

A large number of studies have confirmed that the increase in circulating levels of BCAAs is related to metabolic syndrome and its complications, such as NAFLD, insulin resistance, and type 2 diabetes mellitus (T2DM) (56–58). The complete catabolism of BCAAs in tissues requires many enzymatic steps, most of which occur in mitochondria. BCAA catabolism is regulated by branched-chain amino acid transaminase (BCAT) and branched-chain α-ketoacid dehydrogenase complex (BCKDH) (59). The increase in BCAAs in the lipotoxic environment might lead to mitochondrial dysfunction in the liver, which leads to the impairment of mitochondrial tricarboxylic acid cycle energy during the development of NAFLD (59). Lipotoxicity is a prerequisite for mitochondrial dysfunction caused by BCAAs and might be one of the reasons for the deterioration of insulin resistance in patients with NAFLD.

In addition, the adverse metabolic effects of BCAAs might be mediated by leucine and valine (60). During obesity, excess nutrition increases plasma leucine and valine levels and activates mTORC1 and S6K1. The continuous activation of mTORC1 leads to serine phosphorylation of IRS1 and IRS2, interferes with signal transduction, and targets IRS1 for protein decomposition through the proteasome pathway (61, 62).

Insulin resistance caused by the above mechanisms increases the demand for insulin, and protein degradation might increase the occurrence rate of BCAAs (63). BCAAs promote the stability and nuclear localization of peroxiredoxin 1(Pdx1) in a member ras oncogene family (Rab1A)- and mTORC1-dependent manner and inhibit the transdifferentiation of β cells into α cells, thus playing an important role in regulating insulin resistance (64). Therefore, interfering with the microflora related to BCAA metabolism might be a potential therapeutic target for NAFLD.

#### Tryptophan

Tryptophan (Trp) is an essential amino acid for humans and animals and is found in foods derived from protein, such as meat, milk, nuts, and seeds (65). The tryptophan enzyme is present in the intestinal flora, such as *Bacteroides polymorpha, Clostridium, Enterococcus faecalis*, and *Escherichia coli (* 42), which catalyze the decomposition of dietary Trp and convert Trp into indole and derivatives (66). Indole has a protective effect on the occurrence and development of NAFLD, as it inhibits the proinflammatory activation of macrophages in a PFKFB3-dependent manner, thus reducing the severity of HFD-induced hepatic steatosis and liver inflammation (67).

Indole-3-propionic acid (IPA) is also a tryptophan metabolite produced by intestinal bacteria. Studies have shown that IPA could improve the imbalance in the microflora, increase tight junction proteins in the intestine, and reduce the production of endotoxin. It inhibits NF-κB signal transduction and reduces the levels of proinflammatory cytokines (such as TNFα, IL-1β and IL-6) in response to endotoxin in macrophages to inhibit liver inflammation and liver injury (68). In addition, another tryptophan derivative, indole-3-acetic acid, has been reported to reduce liver adipogenesis (Srebf1, Scd1, PPAR γ, Acaca and Gpam), oxidative stress (ROS and MDA) and inflammation (MCP-1 and TNF-α) to alleviate NAFLD in mice (69, 70).

### Short-chain fatty acids

#### Main components of SCFAs and producting flora

During intestinal digestion, undigested dietary fiber, proteins and peptides are fermented by intestinal flora to form SCFAs. SCFAs are a group of water-soluble free fatty acids with fewer than 6 carbon atoms and are mainly represented by acetic acid, propionic acid, butyric acid, pentanoic acid and so on, of which acetic acid, propionic acid and butyric acid account for more than 95% of SCFAs in the intestine (71). The proximal colon is the site with the highest concentration of SCFAs in healthy bodies (72).

Specifically, acetic acid is the SCFA with the highest concentration in the body and is the center of carbohydrate and fat metabolic pathways. The main acetic acid-producing bacteria are *anaerobes, Bacteroides, Lactobacillus, Streptococcus* and *Bifidobacterium (* 43). The substrate of fermentation is indigestible sugar, including indigestible polysaccharide (NSP) and resistant starch (RS). Propionic acid is mainly produced by *Bacteroidetes* and is the central metabolite of odd-chain fatty acid metabolism, usually produced by the fixed pathway of carbon dioxide (44). Butyric acid is formed by the condensation of acetyl coenzyme A by several specific anaerobes. The main butyric acid-producing bacteria are *Clostridium, Spirillum, Bacillus* and *Ruminococcus* (45, 46). In addition to intestinal fermentation, cellular metabolism, especially fatty aci d oxidation, could also produce SCFAs.In addition, a small amount of isobutyric acid and isovaleric acid will be produced during the catabolism of BCAAs such as valine, leucine and iso-amino acid.

#### The transshipment mode of SCFAs

Acetic acid is a very important raw material for the synthesis of serum total cholesterol (TC) and participates in the hepatic circulation, thus regulating lipid metabolism disorders (73). Propionic acid inhibits the synthesis of TC. Butyric acid has a regulatory effect on inflammation and is used as cell energy and nutrient (74, 75). These effects have led to the association of SCFAs with various lipid metabolic diseases. After being absorbed by the intestine, SCFAs are further used by colon muscle cells or enter the blood circulation and reach other organs. Generally, there are several ways for SCFAs to enter a cell. The first way is passive diffusion. The second pathway is carrier-mediated transport dominated. The third is to activate G protein-coupled cell surface receptor (GPR). SCFAs are not only an important energy source in the body but also represent new signaling molecules that participate in regulating human metabolism by the intestinal flora.

#### SCFAs affect the progression of NAFLD

Several studies have shown that SCFAs affect the progression of NAFLD (76–78). Supplementation with SCFAs can transform the processes in adipose tissue and liver tissue from adipogenesis to fatty acid oxidation (79, 80) and has a protective effect on fatty inflammation induced by a high-fat diet in mice (81). There are obvious changes in fecal microflora during the occurrence and development of obesity-related NAFLD. The feces of patients with obesity or NAFLD are rich in *Proteobacteria, Enterobacteriaceae* and *Escherichia (* 15, 81, 82), but the abundances of some bacteria, such as *Rikenellaceae, Ruminococcaceae, Faecalibacterium* and *Eubacterium*, are reduced (7, 8, 15). The number of *Lactobacillus* and *Bifidobacterium* in the intestinal flora of NAFLD patients decreased; the contents of propionic acid, butyric acid and other metabolites decreased; and the ratio of acetic acid to propionic acid increased (83). After mice with NASH were fed acetate, the steatosis and inflammatory infiltration of the liver were relieved and the TC and triglyceride (TG) contents in the serum and the free fatty acid content decreased (84). Propionic acid has been shown to be related to some biochemical reactions in the body, for instance, inhibiting the rate-limiting enzyme of fat synthesis and enhancing the function of insulin release (77, 85). Butyric acid is related to the expression of proteins in the intestinal tract, which could alleviate the symptoms of liver injury and alleviate inflammation in NASH mice (78).

The effect of SCFAs on NAFLD is mainly achieved through the following two aspects: (1) reducing the inflammatory response and (2) reducing insulin resistance and improving liver steatosis. The most important role of SCFAs in NAFLD is anti-inflammation. The combination of SCFAs and GPR43 causes neutrophil chemotaxis to inflammatory sites and affects the proliferation and function of Treg cells (86). The combination of SCFAs and GPR109A induces the differentiation of Treg cells and IL-10-secreting T cells, thus inhibiting the occurrence of colitis. It has been reported that the combination of SCFAs with GPR43 and GPR109A NOD-like receptor protein 3 (NLRP3) inflammatory bodies (87). The lack of NLRP inflammatory bodies aggravates the disease process of NASH through TLR4 and TLR9 recognition receptors (29). On the other hand, butyric acid and propionic acid may limit the translocation of LPS, reduce the production of proinflammatory cytokines in neutrophils and macrophages after LPS activation, reduce intestinal inflammation and maintain the integrity of the intestinal barrier, thus improving NAFLD (88, 89).

In addition, acetic acid inhibits the secretion of chylous particles and promotes lipid oxidation by upregulating the Adenosine 5’-monophosphate (AMP)-activated protein kinase (AMPK)-peroxisome proliferator-activated receptor γ coactivator 1 α (PGC-1α)-peroxisome proliferator activated receptor alpha (PPARα) pathway (90). SCFAs also increase the expression of GPR41 and GPR43 and promote the secretion of glucagon-like peptide-1 (GLP-1) by L cells. Circulating GLP-1 reaches pancreatic β cells and binds to GLP-1R, thus promoting the release of insulin, reducing insulin resistance and improving hepatic steatosis (91). In patients with steatosis of the liver, there was a decrease in the number of bacteria producing SCFAs and a similar decrease in fecal SCFAs (92); therefore, its effect is weakened accordingly.

#### SCFAs Plays a key role in the main co-morbidity of NAFLD

Furthermore, gut microbiota havs been introduced as a plausible regulator of IL-17A production and functions (93). It has been reported that butyric acid, as a derivative of intestinal flora, could down-regulate the pathological expression of IL-17A (94). The interaction between SCFAs and GPR43 could also regulate the expression of IL-17A (95). It has been reported that, IL-17, released by the visceral adipose tissue, induces eotaxin secretion through the smooth muscle cells present in the atheromatosus vessels to affect the occurrence and development of atherosclerosis (96). Therefore, it is believed that SFCAs could not only regulate NAFLD, but also play an important role in in the main co-morbidity of NAFLD and metabolic syndrome.

### Bile acids

Epidemiological studies have shown that there is a common bile acid pool imbalance in patients with NAFLD, accompanied by changes in specific flora (97). Moreover, it has been confirmed that NAFLD is associated with significant changes in the composition of BAs in the enterohepatic circulation, as well as with the histological characteristics of NASH (1). The higher the proportion of conjugated BAs, the faster is the rate of liver fibrosis (98).

#### BAs affect the composition and abundance of intestinal microflora

There is a complex interaction between BAs and intestinal flora. On the one hand, BAs inhibit the growth of harmful bacteria, affect the number and composition of intestinal flora through their own physiological roles and the mediated signaling pathways, maintain intestinal flora homeostasis, prevent bacterial translocation, and enhance the defense role of the mucosal barrier. BAs regulate the composition of intestinal flora, mainly with an increase in *Firmicutes* and a decrease in *Bacteroides (* 47).

Deoxycholic acid (DCA) was found to increase the F/B ratio of intestinal flora and change the composition of intestinal flora in Apcmin/+ mice treated with DCA. The level of opportunistic pathogens such as *Escherichia coli* and *Shigella* increased significantly, while the abundance of probiotics such as *Lactobacillus* decreased (8). This result shows that BAs can not only change the composition of the intestinal flora but also directly inhibit intestinal flora. As amphiphilic molecules, BAs have lipophilic and hydrophilic properties, which will destroy the phospholipid bilayer, cause cell membrane rupture, and eventually lead to cell death (99).

Through free diffusion, bile acid enters Gram-negative bacteria and causes a stress response, inducing cell RNA to form a secondary structure or causing molecular chaperones such as heat stress shock proteins to denature and lose the ability to function normally, resulting in the failure of normal folding of newly synthesized proteins in bacteria and therefore in bacterial death (100). The antibacterial activity of hydrophobic DCA was 10 times higher than that of cholic acid (CA). Hydrophobic bile acid has a higher affinity for the phospholipid bilayer of the bacterial cell membrane, so it does more damage to the integrity of the cell membrane. BAs oxidize DNA and activate DNA-related repair enzymes (101). BAs chelate with important ions, such as calcium and ferrous ions, inside and outside bacteria, which affects bacterial gene expression and inhibits bacterial movement, reproduction and chemotaxis (101, 102).

#### Effect of intestinal flora on BAs

The intestinal flora affects the synthesis and metabolism of BAs (103, 104). The intestinal flora facilitates the transformation of primary BAs into secondary BAs through a series of enzymatic reactions, which play an important role in BA metabolism. This process includes two steps: (1) uncoupling – some bacteria in the intestinal tract have BA hydrolase (BSH) activity, such as *Lactobacillus, Bifidobacterium, Clostridium* and *Bacteroides*, and under the action of BSH, bound BAs are excreted into the intestinal tract and then catalyzed by BSH to form secondary BAs; and (2) 7 α-dehydroxylation occurs only after uncoupling due to low hydroxyl affinity. The primary BAs, chenodeoxycholic acid (CDCA) and (Cholic acid) CA produce deoxycholic acid (DCA) and lithocholic acid (LCA) after 7 α-dehydroxylation. DCA and LCA are also the most physiologically significant secondary BAs. The main receptors of BAs in regulating host metabolism are Farnesoid X receptor (FXR) and takeda G protein-coupled receptor 5 (TGR5). BAs, as an important signaling molecule, binds to receptors to regulate the inflammatory response and maintain immune homeostasis (105).

Obeticholic, as an agonist of FXR, effectively inhibits the synthesis of bile acid from cholesterol by activating FXR and promoting the expression of fibroblast growth factor (FGF)-15/19 and small heterodimer partner (SHP) (106, 107). In addition, studies have shown that obeticholic could downregulate sterol regulatory element-binding protein-lc (SREBP-lc) and upregulate sirtun 1 (SIRT1) by activating FXR, thus reducing liver fat formation (108). Many clinical trials have shown that as a potent selective FXR agonist, obeticholic improves NASH (109, 110). The mid-term analysis of a phase III clinical study of obeticholic showed that 25 mg/d obeticholic could significantly improve liver fibrosis (111).

In addition, interfering with fatty acid production in other ways can also treat NAFLD. Aramchol is a new compound that binds fatty acids and cholic acid metabolism, which reduces triglycerides and lipid fatty acids by reducing the synthesis of fatty acids. In addition to reducing liver fat, it can also improve insulin resistance. A double-blind placebo-controlled trial involving 60 patients with NAFLD confirmed by liver biopsy (including 6 patients with NASH) showed that 300 mg/d Aramchol reduced liver fat content (112). In addition, as an enteropagin, semaglutide can also improve glucose metabolism and fatty acid oxidation in the liver. The results of a phase II clinical study of semaglutide were reported by the American Association for the Study of Liver Diseases in 2018. Of the 957 patients with NASH, 499 had elevated alanine aminotransferase (ALT). After using semaglutide 0.2~0.4 mg for 54 weeks, 46% of the patients’ ALT levels returned to normal.

### Trimethylamine oxide

Choline comes from exogenous and endogenous sources. Diet provides approximately 70% of the choline, while the rest is synthesized *in vivo*. Choline deficiency hinders the synthesis and secretion of very low density lipoprotein (VLDL) and results in the accumulation of TG in the liver and the pathogenesis of NAFLD; therefore, a choline-deficient diet was applied to develop an NAFLD model in rodents. The intestinal flora converts choline into methylamines, such as trimethylamine (TMA), dimethylamine (DMA) and monomethylamine (MMA), in which TMA further produces trimethylamine oxide (TMAO). A number of studies also showed that the intestinal flora converts dietary components containing choline or TMA structures, such as phosphatidylcholine, betaine, and L-carnitine, to TMA, which enters the liver through the portal vein and rapidly transforms into TMAO under the action of flavin-containing dimethylaniline monoxygenase 3 (FMO3) in the liver (113, 114).

The level of TMAO in the NAFLD group was significantly higher than that in the control group. The level of TMAO was positively correlated with the severity of NAFLD (115). Mechanistically, TMAO upregulates BA synthesis and inhibits BA signal transduction during FXR activation, thus inducing lipogenesis in the liver (116). The gene clusters (CntA, CntB) are responsible for the production of TMA are commonly found in *obligately anaerobic Clostridia* (*phylum Firmicutes)* and *facultatively anaerobic Enterobacteriaceae* (*phylum Proteobacteria*) *(* 48, 49), and the abundance of the latter group in the feces of individuals fed a high-fat diet is significantly increased (117, 118).

This increase might be related to the low-grade mucosal inflammation induced by a high-fat diet and the mitochondrial bioenergy causing dietary damage in the colonic epithelium (119). The increase in Proteus in patients with NAFLD was also reported to be related to the increased production of TMA and TMAO (120). Conversely, a study showed that the intestinal microbial metabolite TMAO restores the diversity of intestinal flora, inhibits intestinal cholesterol absorption, reduces liver cholesterol overload, and thus reduces cholesterol-induced endoplasmic reticulum (ER) stress and cell death in the liver (121). Currently, research on TMAO is still very limited, and in-depth studies are needed to understand the precise role of intestinal flora imbalance and its related TMAO in NAFLD.

## Targeting intestinal flora to treat and prevent NAFLD

### Diet

There are no approved drugs available for NAFLD treatment at present, and lifestyle intervention, including dietary restrictions, a Mediterranean diet and a low-carbohydrate diet (LCD), is considered to be the main treatment for NAFLD. Reasonable diet planning and lifestyle changes could improve the composition of intestinal flora and reduce the risk of NAFLD.

The study found that a combination of a Mediterranean diet and LCD significantly reduced the liver fat content and cardiovascular metabolic risk parameters (122). The Mediterranean diet reduces the abundance of *Escherichia coli* and increases the abundances of *Bifidobacterium* and *Purkinje*, thereby modifying the intestinal flora to yield a healthier state (123). The Mediterranean diet includes whole grains and monounsaturated fatty acids. Fiber and polyphenols in whole grains reduce energy intake; increase *Bifidobacterium, Lactobacillus* and *Clostridium* in the intestinal tract; and increase butyric acid in the intestinal tract, thus reducing insulin resistance and exerting an anti-inflammatory effect to improve NAFLD (124).

LCD refers to a diet that limits carbohydrates (energy supply ratio < 45%), increases fat and protein, and reduces the intake of refined grains and added sugar (125, 126). Adil and other studies have shown that LCD intervention in obese people increases the abundance of *Streptococcus* and *Lactococcus*, resulting in increased folic acid biosynthesis and upregulation of the fatty acid degradation pathway (127). Therefore, the interaction between an LCD and intestinal flora might help to explain the diet-associated anti-inflammation and lipid-lowering effects in the liver.

### Endurance exercise

The beneficial effects of exercise on improving intestinal flora have been widely proven in rehabilitation medicine and sports science. The evidence shows that proper exercise significantly changes the structure of the intestinal flora to improve health status (128). Study has shown that rotational exercise increases the number of *Bifidobacterium* and *Lactobacillus* in rodents (129). *Bifidobacterium* is one of the most important physiological bacteria in human and animal intestines. It has become a potential treatment for NAFLD because of its anti-inflammatory, antioxidant, regulation of gastrointestinal peristalsis and other effects (130).

In addition, experiments were conducted on obese and thin subjects, and it was found that exercise training could cause changes in the intestinal flora and increase the number of butyrate-producing bacteria, but this change depends on the change in body mass index (131, 132). It has been demonstrated that butyric acid increases insulin sensitivity, regulates inflammatory cytokines and lipid metabolism, and reduces liver injury, fibrosis progression, and intestinal barrier dysfunction, thus improving NAFLD (133). The above results suggest that exercise changes the abundance of intestinal flora, and this effect is partly independent of the effect of diet. However, few studies have directly linked the beneficial effects of exercise intervention on NAFLD through ameliorating intestinal microorganism composition.

### Microecological therapy

In the treatment of some diseases, microecological therapy has become a potential therapy to maintain the health of the host (134, 135). As the main microecological regulators, probiotics play an important role in maintaining the health of the host by regulating the structure of the intestinal flora. Probiotics include different kinds of bacteria that regulate intestinal flora, enhance intestinal barrier function, alleviate immune and metabolic damage (136), reduce the systemic inflammatory response, and upregulate fatty acid oxidation (137). Probiotics can also reduce cholesterol levels, liver steatosis and its associated inflammation (138). In addition, probiotics improve liver cholesterol and lipid metabolism by improving SCFAs and BAs metabolism (136, 139) and liver fibrosis (140). A meta-analysis confirmed that probiotics improve the level of ALT、aspartate aminotransferase (AST) and gamma glutamyl transferase (GGT) in patients with NAFLD (141). In a randomized controlled trial of 42 patients with NAFLD, fasting blood glucose, insulin resistance, TNF-a and IL-6 were significantly decreased after 8 weeks of probiotic intervention (142).

However, although probiotics have been proposed for the treatment and prevention of obesity-related NAFLD patients, their therapeutic uses are not supported by high-quality clinical studies (143). In addition, some studies hold opposite views on the role of probiotics in NAFLD. For instance, there is study has shown that probiotics reduce liver lipid accumulation by reducing intestinal permeability and inhibiting chronic inflammation without significantly changing the composition of the intestinal flora (144). Other results showed that taking probiotics for one year changed the fecal microbiome of the patients, but did not reduce the liver fat content and markers of liver fibrosis (145). Moreover, the molecular mechanism linking the beneficial effect of probiotics in NAFLD has not been precisely identified. Up till now, the clinical research on probiotics in the treatment of NAFLD is still limited. To further explore the specific efficacy of probiotic therapy on NAFLD and its possible mechanism, more clinical and basic studies are needed.

### Antibiotic treatment

The use of antibiotics has a significant effect on intestinal flora (146). Animal studies have shown that antibiotics rapidly and significantly change the composition of intestinal flora. Antibiotics (ampicillin, neomycin, metronidazole, and vancomycin) reduce the liver inflammatory response by regulating the level of free and bound secondary BAs (147). In addition, some studies have shown that antibiotics reduce hepatic steatosis by inhibiting intestinal FXR, thereby downregulating the expression of sterol regulatory element-binding transcription factor 1 (SREBP1C) and cell death-inducing DFFA like effector A (CIDEA) in the liver (148). A study also revealed that antibiotics reduce liver inflammation and the NASH phenotype by inhibiting the activation of hepatic migratory macrophages (149).

However, antibiotics have the most destructive and lasting effect on the diversity, structure and function of the intestinal flora (150). Therefore, the use of antibiotics might have some negative effects. On the one hand, the use of antibiotics will lead to an imbalance in the diversity of the intestinal flora, with a lack of beneficial *Bifidobacterium* and *Clostridium stenosum* (151) and an increase in the pathogenic bacteria *Enterobacteriaceae, Enterococci* and *Staphylococci (* 152). On the other hand, the use of antibiotics will affect the content of SCFAs, which are metabolites of the intestinal flora. SCFAs are closely related to the occurrence and development of a variety of diseases, such as inflammatory bowel disease, type 1 diabetes and NAFLD (153).

In addition, overuse of antibiotics in clinical practice is responsible for the increase in the incidence of gastrointestinal diseases (154). Antibiotic resistance is increasing worldwide and poses a fundamental and long-term threat to human health. Even short-term courses of antibiotics are related to the development of drug-resistant bacteria in the human intestinal tract. In addition, some studies have shown that penicillin G (Pen G) and erythromycin (Ery), especially the latter, aggravate lipid deposition and the inflammatory response in the liver (155, 156). Current studies have revealed that the use of antibiotics is a double-edged sword in the treatment of NAFLD.

### Fecal microbiota transplantation

Fecal microbiota transplantation (FMT) is a new treatment strategy for diseases related to intestinal microecological imbalance. The principle is to reintroduce or establish a stable environment that affects the endogenous bacteria and the host by using intestinal flora from healthy donors by enema, oral capsule or endoscopy (157). After FMT treatment, the bacterial state provided by the donor could be maintained in the intestinal cavity of the patient for 2 weeks to 1 month (158). FMT has the following advantages (1): the species of transplant flora are rich (2); the number of transplant flora is large; and (3) the original functional bacteria are retained to the maximum degree. Therefore, FMT significantly improves the disorder of intestinal bacteria and is currently recognized as the most effective method for restoring the balance of intestinal microecology (159).

FMT in mice with metabolic syndrome could increase the abundance of beneficial flora and reduce the abundance of harmful flora, and the therapeutic effect of FMT on NAFLD has been positively demonstrated in many animal and clinical studies (160–162). It was found that the transplantation of fecal bacteria from mice fed a normal diet could significantly reduce the triglyceride content in the livers of mice fed a high-fat and high-sugar diet and alleviate the progressive deterioration of the liver histology. In addition, fecal bacteria transplantation could partially correct the imbalance in intestinal flora in high-fat and high-glucose mice, increase the butyrate concentration in feces (161), and significantly alleviate the degree of endotoxemia, liver steatosis and inflammatory necrosis in NAFLD models (161).

However, there are risks of pathogen infection and colonization resistance in FMT (163, 164). At present, there is still a lack of clinical research on the effect of fecal bacteria transplantation on human metabolic syndrome and NAFLD. In addition, although fecal donors and samples transplanted with fecal bacteria have been tested for a variety of potentially pathogenic bacteria, viruses, parasites and other microorganisms, the complete microbial composition of the sample to be transplanted cannot be determined. Therefore, many scholars have raised concerns about the safety of fecal bacteria transplantation in humans. According to the statistics of 7562 published articles by Sinan Wang et al. in 2016, there are 78 kinds of side effects and adverse reactions related to FMT, such as fever, vomiting, gastrointestinal spasm and tachycardia, with an incidence of 28.5% (165). FMT has different implementation protocols in different institutions, and there is no standardized guidance for FMT worldwide.

## Discussion

In the past 15 years, a large number of studies have found that there are a vast number of microflora in the intestinal tract of the body, including bacteria, viruses and fungi. Microflora can form symbioses with the host, and the maintenance of their homeostasis guarantees human health. The intestinal microflora has become an important regulator of host energy metabolism and substrate metabolism (166–168). A “biological imbalance” in the intestinal flora is generally considered to be a disruption in the diversity and composition of microbiota, which is related to the occurrence of intestinal and parenteral inflammation, immunity and other related diseases, including NAFLD (29). In an in-depth study, it was found that NAFLD patients have an intestinal flora imbalance; for example, the abundances of *Proteobacteria* and *Enterobacter* are increased, while the abundances of *Ruminococcus* and *Firmicutes* are decreased. With the progression of NAFLD to advanced liver fibrosis, the number of Gram-negative bacteria is increased, especially *Proteobacteria (* 124).

The abundance of bacteria in human intestinal flora is related to the occurrence of NAFLD, and the changes in intestinal flora related to it mainly depend on the stage of development of the disease (120). The most typical general characteristics of NAFLD development include a decrease in intestinal flora diversity, an increase in the number of Gram-negative bacteria (mainly *Proteobacteria*) and a decrease in the number of Gram-positive bacteria (mainly *Spirochaetes*) *(* 8, 169, 170). The leading flora with respect to the composition of the intestinal flora is also changed from beneficial flora to harmful flora, which leads to intestinal inflammation and the production of metabolic toxins, thus causing intestinal barrier dysfunction, exposing the liver to flora metabolites and promoting the development of NAFLD (120).

In the intestinal flora of NAFLD patients, the abundances of *Firmicutes* and *Proteobacteria* are increased; these phyla metabolize choline to produce TMA, which reduces the bioavailability of choline, thereby reducing the synthesis and release of very-low-density lipoprotein, while TMA increases insulin resistance and promotes fatty acid uptake by the liver after oxidation (171, 172). The increased abundance and excessive proliferation of intestinal conditional pathogenic bacteria such as *Escherichia, Dysgonomonas* and *Bilophila* promote the production of endotoxin and endogenous ethanol, thus aggravating the inflammatory reaction and promoting insulin resistance. At the same time, the abundances of beneficial bacteria decreased, which normally inhibits the production of SCFAs, impaired its ability to improve NAFLD, including maintain the integrity of the intestinal mucosal barrier, reduce the content of harmful microflora and inhibit inflammation (7, 19, 20).

A brief review of the mechanism summarized above shows that the destruction of the intestinal epithelial barrier caused by intestinal flora imbalance is an important condition for the development of NAFLD and NASH. The dysfunctional microflora destroys the integrity of intestinal mucosal barrier function through endotoxins, which translocate into the liver, resulting in fat accumulation, activation of inflammatory cytokines and the accumulation of endotoxins. The imbalance in the intestinal flora might also include regulating the inflammatory response through flora metabolites, regulating TLR signaling, and changing the balance between regulatory and proinflammatory T-cell subsets, thus affecting the host immune system (173). Intestinal flora disorder also affects the metabolic system, including changes in BAs composition, the production of SCFAs from dietary fiber, and the conversion of choline to TMA, thus leads to the disorder of glucose and lipid metabolism, including insulin sensitivity and hepatic steatosis. Therefore, regulation of the intestinal flora to affect the metabolism and immune signal transduction of susceptible hosts might be a potential target for the treatment of metabolic syndrome and NAFLD.

For patients with NAFLD, treatments for intestinal flora, such as probiotics and microecological therapy, have made good progress in some studies. Emerging treatments, such as FMT, are also being actively explored. However, to date, the results have been limited, and there are some side effects. Therefore, more clinical studies are needed to evaluate their efficacy.

In this review, it is suggested that the intestinal flora plays an important role in the onset and progression of NAFLD through its effects and its metabolites and is a key target in the treatment of NAFLD. The pathways regulated by intestinal flora are intricately related. Changes in the composition and proportion of the intestinal flora will cause an imbalance in positive and negative feedback mechanisms, which will affect the occurrence and development of NAFLD. With the development of research methods and an in-depth understanding of the intestinal flora, the precise role and mechanism of different microflora in the progression of NAFLD can be further explored to provide therapeutic targets.

## Author contributions

JL and AW wrote the manuscript. JC provided valuable suggestions and guided the manuscript. Z-GS and HL contributed equally to supervise the project and edit the final manuscript. All authors have approved the final version of this paper.

## Funding

This work was supported by the National Science Foundation of China (81770053, 81970364 to Z-GS, 81870171, 82170436 to JC) and grants from the Hubei Province Innovation Platform Construction Project (20204201117303072238 to HL)

## Conflict of interest

The authors declare that the research was conducted in the absence of any commercial or financial relationships that could be construed as a potential conflict of interest.

## Publisher’s note

All claims expressed in this article are solely those of the authors and do not necessarily represent those of their affiliated organizations, or those of the publisher, the editors and the reviewers. Any product that may be evaluated in this article, or claim that may be made by its manufacturer, is not guaranteed or endorsed by the publisher.
